# Biometry and visual function of a healthy cohort in Leipzig, Germany

**DOI:** 10.1186/s12886-016-0232-2

**Published:** 2016-06-07

**Authors:** Maria Teresa Zocher, Jos J. Rozema, Nicole Oertel, Jens Dawczynski, Peter Wiedemann, Franziska G. Rauscher, For the EVICR.net

**Affiliations:** Department of Ophthalmology, Leipzig University Hospital, Liebigstrasse 10-14, 04103 Leipzig, Germany; Department of Ophthalmology, Antwerp University Hospital, Wilrijkstraat 10, 2650 Edegem, Belgium; Department of Medicine and Health Science, University of Antwerp, Universiteitsplein 1, 2610 Wilrijk, Belgium

**Keywords:** Ocular biometry, Visual function, Gullstrand, Cross section, Dioptric distance

## Abstract

**Background:**

Cross-sectional survey of ocular biometry and visual function in healthy eyes across the life span of a German population aged 20 to 69 years (*n* = 218). Subject number in percent per age category reflected the percentage within the respective age band of the population of Leipzig, Germany.

**Methods:**

Measurements obtained: subjective and objective refraction, best-corrected visual acuity, accommodation, contrast sensitivity, topography and pachymetry with Scheimpflug camera, axial length with non-contact partial coherence interferometry, and spectral-domain optical coherence tomography of the retina. Pearson correlation coefficients with corresponding *p*-values were given to present interrelationships between stature, biometric and refractive parameters or their associations with age. Two-sample *T*-tests were used to calculate gender differences. The area under the logarithmic contrast sensitivity function (AULCSF) was calculated for the analysis of contrast sensitivity as a single figure across a range of spatial frequencies.

**Results:**

The results of axial length (AL), anterior chamber depth (ACD) and anterior chamber volume (ACV) differed as a function of the age of the participants (rho (p value): AL −0.19 (0.006), ACD −0.56 (< 0.001), ACV-0.52 (< 0.001)). Longer eyes had deeper ACD (AL:ACD 0.62 (< 0.001), greater ACV (AL:ACV 0.65 (< 0.001) and steeper corneal radii (AL:R1ant; R2ant; R1post; R2post 0.40; 0.35; 0.36; 0.36 (all with (< 0.001)). Spherical equivalent was associated with age (towards hyperopia: 0.34 (< 0.001)), AL (−0.66 (< 0.001)), ACD (−0.52 (< 0.001)) and ACV (−0.46 (< 0.001)). Accommodation was found lower for older subjects (negative association with age, *r* = −0.82 (< 0.001)) and contrast sensitivity presented with smaller values for older ages (AULCSF −0.38, (< 0.001)), no change of retinal thickness with age. 58 % of the study cohort presented with a change of refractive correction above ±0.50 D in one or both eyes (64 % of these were habitual spectacle wearers), need for improvement was present in the young age-group and for older subjects with increasing age.

**Conclusion:**

Biometrical data of healthy German eyes, stratified by age, gender and refractive status, enabled cross-comparison of all parameters, providing an important reference database for future patient-based research and specific in-depth investigations of biometric data in epidemiological research.

**Trial registration:**

ClinicalTrials.gov # NCT01173614 July 28, 2010

## Background

The optics of the human eye is based on the refractive parameters of the individual ocular structures, each of which is affected differently by age. Well known examples of this are the thickening of the crystalline lens with aging and the gradual flattening of the anterior chamber [1]. Although normal aging has been studied extensively in the literature, some studies limit themselves to subsections of the population, such as subjects over 40 years, children, certain nationalities or ethnicities, or emmetropes [2–4]. Other studies presented only certain parameters of ocular biometry, or concentrated on prevalence of specific eye conditions (e.g., cataract, AMD) [5, 6]. This study concentrates on various biometric parameters in connection with refraction and retinal measurements with optical coherence tomography (OCT) in a healthy population. Although measurements in healthy eyes across the life span could provide an invaluable reference in the form of a normative database for a multitude of ocular biometric factors, few studies have presented recent data for a European population since many of the population − based studies have been conducted in developing countries [7–9]. In Europe, three larger population-based studies have focused on the aspects of biometric data to determine the prevalence, incidence and major risk-factors of age-related macular degeneration, glaucoma and diabetic retinopathy in adults over 35 years [10–14]. Results on refractive error and related ocular biometry were presented for British adults over 48 years of age [15].

As those previous eye studies in Europe concentrated on older eyes and on the prevalence of ocular pathologies, the current study aimed to obtain a full cross-section of a healthy population using modern measuring devices to establish reliable reference values for future work. A specific strength of this study is the investigation of many different biometric values together with detailed information on refraction and retinal properties. OCT was implemented to eliminate subjects with retinal changes or pathologies from the sample. Based on these data differences in parameters across the different age groups can be determined, and it provides reference values stratified by age range, gender and refractive status. This may help to find inter − correlations of optical parameters in the human eye.

## Methods

### Study population

The cohort presented in this work was measured under the framework of “Project Gullstrand” (Ethics Committee of the Antwerp University Hospital (No. B30020072406)), a multi-centred cross − sectional study performed at different European clinical sites [16, 17]. The Leipzig data-set, for the first time, presents various biometric measures in a group of healthy German subjects with strictly examined absence of degenerative changes of the retina. This cohort was recruited to mirror the distribution of age and gender of the population of Leipzig (Saxony, Germany) [18]. The data presented here were obtained between July and December 2011. The study adhered to the Tenets of the Declaration of Helsinki and approval for the study was obtained from the Ethics Committee of the Medical Faculty of the University of Leipzig (No. 162/11) and is registered as ClinicalTrials.gov # NCT01173614.

Subjects were recruited through public announcements and press releases. However, in a first telephone interview the interested subjects were asked a series of health related questions in order to exclude subjects based on the inclusion criteria (age 20–69 years old, ametropia between −10 D and +10 D) before examinations, and the exclusion criteria, which were prior ocular surgery, amblyopia, refraction larger than ± 10 D, corneal or retinal pathologies in either eye, systemic diseases (e.g., diabetes mellitus, hypertension, multiple sclerosis, Grave's disease, …), pregnancy of more than 5 months at the moment of testing, as well as recent wear of hard contact lenses. Likewise, a 2-day break prior to testing was required with soft contact lens use.

### Examination procedures

After informed consent was obtained, an interview was conducted to determine ocular and medical history, level of education and height and weight as self − reported by the subject. Then subjects were asked to fill out the 25 item National Eye Institute visual functioning questionnaire (NEI − VFQ − 25), developed at RAND under the sponsorship of the National Eye Institute [19, 20].

The following refraction parameters were used following the uniform method for visual acuity notation in scientific publication [21]. Refraction was estimated with an autorefractometer (Automatic Refractor/Keratometer Model 599, Humphrey Instruments, Carl Zeiss Meditec AG, Jena, Germany). A single operator then performed a non − cycloplegic subjective refinement of the refractive correction until the best corrected visual acuity (BCVA) was obtained. Uncorrected and corrected distance visual acuity (UDVA, CDVA) were measured monocularly and binocularly with a trial frame at 4 m, using an ETDRS chart [22], which was housed in a light box. Participants who failed to read the largest letter unaided at 4 m were retested at 2 m then at 1 m. Testing began with the first letter on the top row. When having difficulty reading a letter, the subject was encouraged to guess. Visual acuity was scored as the total number of letters read correctly in logMAR units (logarithm of the minimum angle of resolution). The full refraction was noted and for further analysis the spherical equivalent (SE) or the dioptric distance (described below, [23]) were used as measures of refractive error.

After determining the best CDVA, accommodation was tested for each eye separately and binocularly using the negative lens test with an ETDRS chart at 4 m distance, while wearing the distance correction [24]. The subject was asked to focus two lines above the lowest line still readable with best correction. A lens of −10 D was placed in front of the eye and the subject was asked if he or she could still read the line. If this was not the case, the same was repeated with a lens of 0.5 D lower power until the subject was able to see the line clearly again.

Contrast sensitivity was measured with the Visual Contrast Test System (VCTS; Vistech Consultants, Dayton, USA) panel in a room with 80–100 lux illuminance. Before, the pupil size was determined with a pupil size gauge. This contrast sensitivity panel uses a grid of contrast levels for a range of spatial frequencies, which allows a rough reconstruction of the entire contrast sensitivity curve. With the VCTS panel the contrast increases from left to right and the spatial frequency from top to bottom. Each column contains gratings with spatial frequencies of 1.5, 3, 6, 12 and 18 cycles per degree (cpd). Each line contains 8 grids with progressively decreasing levels of contrast and a uniform grey field at the end of each line. The subject was seated at a distance of 2 m from the panel and had the task of describing the orientation of the lines of the respective grating being either vertical or 15° to the right or to the left. When the contrast of a grid has fallen below the threshold value, only a single grey field is perceived. The last grating of each row that was identified correctly was noted. The testing was carried out monocularly and binocularly with optical correction, if applicable.

Topography and pachymetry were measured with a Pentacam Scheimpflug camera (Oculus Optikgeräte GmbH, Wetzlar, Germany). Using non − contact partial coherence laser interferometry (IOL Master; Carl Zeiss Meditec AG, Jena, Germany), five measurements for axial length (AL) were obtained and the mean of these measurements as calculated by the IOL Master was noted in the datasheet. AL was measured as the distance from the anterior corneal vertex to the retinal pigment epithelium along fixation, automatically adjusted to the distance to the internal limiting membrane as used as a reference plane in ultrasound techniques [25]. The displayed results of the axial length measurements are therefore compatible with immersion ultrasound measurements through the use of an internal, statistically verified calculation algorithm [26, 27].

Further measurements were carried out employing optical coherence tomography (OCT) (Spectralis OCT, Heidelberg Engineering) to measure retinal thickness in the foveal region. The OCT was also useful in detecting diseases of the retina or pre − clinical abnormalities. OCT instrumentation and imaging technique have been described in detail elsewhere [28–31]. The central retinal thickness was defined as the distance between the internal limiting membrane to the outer border of the retinal pigment epithelium via the automatic segmentation algorithms of the Spectralis software by which the macular region is sectioned into three circular rings (1 mm, 3 mm and 6 mm diameter) which are subdivided into four quadrants to form nine regions of analysis. An average retinal thickness and retinal volume can be reported for central, superior inner, inferior inner, temporal inner, nasal inner, superior outer, inferior outer, temporal outer, nasal outer regions. The average of all points within the inner circle of 1-mm diameter is defined as central foveal subfield thickness (CFST). With accurate centration, the central foveal subfield (1 mm) includes the foveal minimum. The minimum retinal thickness is defined as minimal central retinal thickness (CRTmin).

### Calculations and statistical analysis

Statistical analysis was performed using Minitab 14 (Minitab Inc., State College, Pennsylvania, USA). Due to the high degree of correlation between the eyes of a subject, only data of the right eye are presented in this study. All variables were first analysed by calculating the mean, standard deviation (SD) and 95 % confidence interval (95 % CI) after tests for normality using Q-Q-plots [32]. Further, mean and SD of the variables was calculated for age decades. Pearson correlation coefficient rho (r) and its corresponding p-value was calculated to present the interrelationships between stature and biometric and refractive parameters. Labeling systems exist to roughly categorize r values where correlation coefficients (in absolute value) of ≤ 0.35 are generally considered to represent low or weak correlations, 0.36 to 0.67 reflect modest or moderate correlations, and 0.68 to 1.0 identify strong or high correlations, and r coefficients of ≥ 0.90 represent very high correlations [33]. This notation was used throughout the manuscript. Meaningful clinical relevance of such associations should be established by calculating the coefficient of determination. It is obtained by simply squaring the correlation coefficient rho. R^2 is defined as the percent of the variation in the values of the dependent variable (y) that can be explained by variations in the values of the independent variable (x). This presents an index for the strength of an association, a value of R2 ≥ 50 % (rho > 0.7) can be considered a relevant correlation [34]. Two-sample *T*-tests were used to calculate gender differences (*p*-values stated without Bonferoni correction, based on the planned outline of the procedure). ANOVA analysis with post-hoc *t*-tests were not carried out to investigate possible differences for individual parameters between age-categories (20–29, 30–39, 40–49, 50–59, 60–69 years of age) stated in Table 1, as a correlation with age itself is given for each biometric parameter measured as part of the result section.

**Table 1:** “Subjective refraction data stratified by gender and age”

	Women					Men				
	*n*	Sphere [D]	Spherical equivalent [D]	Dioptric Distance to habitual correction [D]	Dioptric Distance to a 0.00D lens [D]	*n*	Sphere [D]	Spherical equivalent [D]	Dioptric Distance to habitual correction [D]	Dioptric Distance to a 0.00D lens [D]
20–29 years	24	−0.85 ± 1.64	−0.99 ± 1.64	0.45 ± 0.27	1.23 ± 1.48	26	−1.07 ± 1.39	−1.46 ± 1.46	0.43 ± 0.26	1.78 ± 1.16
30–39 years	19	−1.34 ± 2.06	−1.63 ± 2.17	0.32 ± 0.29	2.06 ± 1.79	20	−1.62 ± 2.29	−2.00 ± 2.35	0.46 ± 0.36	2.07 ± 2.31
40–49 years	32	−0.86 ± 1.93	−1.13 ± 2.00	0.50 ± 0.35	1.33 ± 1.90	29	−0.24 ± 2.13	−0.56 ± 2.11	0.41 ± 0.33	1.39 ± 1.75
50–59 years	19	+0.91 ± 1.22	+0.74 ± 1.17	0.52 ± 0.43	1.15 ± 0.79	19	0.16 ± 2.36	−0.13 ± 2.41	0.70 ± 0.50	1.64 ± 1.76
60–69 years	16	+0.63 ± 1.94	+0.24 ± 2.18	0.73 ± 0.36	1.68 ± 1.47	14	+1.07 ± 1.66	+0.68 ± 1.76	0.55 ± 0.17	1.27 ± 1.06
All	110	−0.42 ± 1.95	−0.66 ± 2.02	0.49 ± 0.35	1.45 ± 1.56	108	−0.47 ± 2.15	−0.81 ± 2.18	0.49 ± 0.35	1.64 ± 1.67

**Fig. 1:**
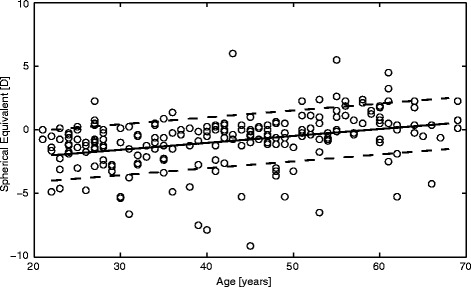
“Spherical equivalent by age of subject (*n* = 218)”. Caption: Scatterplot of age and spherical equivalent for subjective refraction. In the study population there was weak association between age and subjective refractive error (*r* = 0.335, *p* < 0.001). Regression equation: Spherical equivalent = −3.03 + 0.054 Age; 50 % confidence interval: 1.96 D

Caption: Mean (± standard deviation) of sphere, spherical equivalent (SE) and dioptric distance (DD) to a 0.00D lens and dioptric distance of habitual spectacle correction to new subjective refraction, determined by best corrected visual acuity per age decade for women and men. SE (subjective) for the right eyes of 20–29 year olds was found to be −1.24 ± 1.55 D; 30–39: −1.82 ± 2.24 D; 40–49: −0.86 ± 2.06 D; 50–59: +0.31 ± 1.92 D; 60–69: +0.45 ± 1.97 D, see also Fig. 1. The hyperopic shift resulted in more emmetropic eyes in the 40–49 years decade, followed by a higher percentage of hyperopic eyes from 50 years onwards: 20–29: H = 4 %, E = 34 %, M = 62 %; 30–39: H = 8 %, E = 31 %, M = 62 %; 40–49: H = 8 %, E = 52 %, M = 39 %; 50–59: H = 45 %, E = 26 %, M = 29 %; 60–69: H = 50 %, E = 30 %, M = 20 % (% rounded to present full numbers). It is possible that some of the differences found between younger and older age groups may reflect other factors (e.g., changes in prevalence of refractive error) and therefore differences in refractive error observed may not properly account for changes in refractive error over time

The dioptric distance to habitual correction specifies the average deviation of the subject’s habitual corrective lens (or no correction in-situ) to the optimum spectacle correction. The deviation of the habitual corrective state to its optimal corrective state, identified as part of the study, increased with increasing age and was greatest for older subjects. A second at-risk group for malcorrection was identified in the 20–29 age bracket, where about half a dioptre blur was measured

The mean and SD of contrast sensitivity values in log units by age and gender are provided. To obtain log units from the contrast sensitivity level, a value key for the Vistech VCTS 6500 contrast sensitivity test system was used and log units of these values were calculated [35]. For further analysis of the contrast sensitivity as a single figure across a range of spatial frequencies, the area under the logarithmic contrast sensitivity function (AULCSF) was calculated according to the method of Applegate and colleagues [36]. In brief, the AULCSF was calculated by integrating a third order polynomial fitted to the log contrast sensitivity data between the fixed limits of 0.18 (corresponding to 1.5 cpd) and 1.26 (18 cpd) on the log spatial frequency scale; based on the raw data supplied by the test employed. It is, however, also possible to employ linear interpolation and integration between 1.5 and 18 cpd to compute an area under the contrast sensitivity curve [37]. Such a single-index criterion represents contrast sensitivity data as one number and therefore facilitates comparison and statistical analysis.

For further analysis, data were analysed by refractive status categorised by spherical equivalent (SE; sphere plus half cylinder based on sphere (S) and cylinder (C)) or categorised by dioptric distance (described below). Separation by SE was done by division into three groups (myopia (M), emmetropia (E) and hyperopia (H)). Myopia was defined as a SE less than −0.5 D and hyperopia as a SE greater than +0.5 D. For facilitation of sub-analysis of future publications, the data are also given as five subcategories, to indicate that there is a considerable functional difference between uncorrected eyes with a refraction smaller than ±2D and eyes with refractive error larger that ±2D. ±2D also roughly corresponds to the emmetropic peak of the refraction distribution. Tables 5, 6 and 7, as a reference: manifest myopia (< −2D), low myopia (−2D ≤ −0.5D), emmetropia (−0.5D ≥ ≤ +0.5D), low hypermetropia(+0.5D ≥ +2D) and manifest hypermetropia (> +2D).

Where possible, the mean difference between subpopulations was given (e.g., difference between men and women for various age groups amounting to an overall mean difference for men and women). However, other publications provide only the overall mean (e.g., mean of all women irrespective of age), therefore some cross − referencing in our study was only possible by computation based on values given in another investigation. This comparison (i.e., men and women) of averaged data, is referred to as difference of the mean(s) to distinguish the difference.

Description of refractive error by means of spherical equivalent is widely employed by ophthalmologists and optometrists. However, in a mathematical description of ophthalmic lenses, it is more suitable to employ matrix formalism [23, 38], which enables an accurate derived measure of every full refraction as a single term, making it independent of unit conversion problems otherwise present. While sphere and cylinder refer to power along principal directions, power components refer to fixed coordinate axes and are grouped into a matrix. The dioptric power matrix, F, is defined as

$$ \mathrm{F}=\left(\begin{array}{cc}\hfill S+C\  sin \mathit{^2}\ \alpha \hfill & \hfill -C \cos \alpha \sin \alpha \hfill \\ {}\hfill -C \cos \alpha \sin \alpha \hfill & \hfill S+C\  cos \mathit{^2\alpha}\hfill \end{array}\right) $$, and it accounts for all paraxial properties of the ophthalmic lens (prismatic effects are not considered here). In order to determine the mean refractive status for the study population, data for sphere, cylinder, and axis (α) measured by subjective refraction were transformed into a dioptric power matrix. In order to compare measures of refraction (e.g., old and new refraction), the dioptric distance (DD) was used. The dioptric distance DD is defined as the distance in the power domain between lenses with different astigmatic effects. This distance between two points based on the Frobenius norm, is

$$ \mathsf{D}\mathsf{D} = \sqrt{0.5\ \left({\left(Fxx1-Fxx2\right)}^2 + {\left(Fyy1-Fyy2\right)}^2 + 2{\left(Fxy1-Fxy2\right)}^2\right)} $$

with Fxx, Fyy, Fxy as the elements of the matrix. The factor of 2 results from the equal diagonal elements of the matrix as depicted above. The prefactor 0.5 is conveniently employed in order to scale the dioptric distance to compare data logically (i.e., the dioptric distance of two spherical refractions equates to the distance between spheres).

Furthermore, the overall refractive error of the study population was provided by calculating the mean dioptric distance to a 0.00 D lens, as this transfers each measured refraction into a single number which allows such averaging. Additionally, the mean dioptric matrix and the mean dioptric distance were determined for different age decades. The dioptric distance to a 0.00 D lens results in a simplification of the above formula, as the second terms are replaced by zero.

$$ \mathrm{D}\mathrm{D}=\sqrt{{\left(S+\frac{C}{2}\right)}^2+\frac{C^2}{4}} $$

In order to establish how many subjects were in need of a new correction, a change in SE of 0.50 or a visual acuity improvement of one line of the ETDRS chart (equal to 0.1 logMAR) is commonly used [29, 39−41]. A change of 0.50 D for SE was employed to calculate differences between habitual correction and best corrected refraction. Differences are given as percentages above this criterion to indicate potential need of improved correction. For dioptric distance such a cut − off criterion for when a new correction would be required, needed to be similarly established. Here, for practical reasons, a dioptric distance between old corrective state and new best corrected refraction of 0.35 D was chosen for previous spectacle wearers and a dioptric distance of 0.50 D was chosen for non-spectacle wearers. This threshold of change of 0.35 D (and 0.50 D) is above the known diurnal fluctuation [42] in refractive status and therefore underlining a true change in refraction. A dioptric distance of 0.35 D is equivalent to a change in sphere of 0.35 D or equivalent to a change in cylinder of 0.50 D, both of which constitute a similar influence on visual acuity. The dioptric distance of 0.50 D is equivalent to a change in sphere of 0.50 D or a change in cylinder of 0.71 D, again both affecting visual acuity to the same extent. The effect on visual acuity is based on previous research showing that the influence on vision is the same for refractions resulting in the same dioptric distance [43].

## Results

Of the 245 participants eligible for participation based on the telephone interview, 27 subjects had to be excluded because it was established during examination that they did not fulfil the inclusion criteria (amblyopia (7), previously unknown health problems (4, diabetes mellitus, arterial hypertension, multiple sclerosis and glaucoma), rigid gas permeable lens wear (3) and pathologies of the cornea or retina as established by eye examination and OCT (13)), leaving 218 Caucasian subjects for the analysis. The data presented are based on 108 men and 110 women aged 21–69 years with mean ± SD age of 42 ± 13 years and 43 ± 13 years, respectively. See Fig. 1 for distribution of age across the sample.

The aim of this investigation is to establish normal sample data of biometric measurements based on strict inclusion criteria. The study cohort was specifically recruited to reflect the age and gender distribution of the population of Leipzig and is therefore unbiased by a specific selection [18]. In order to mirror this distribution for the final sample, (i) age and gender brackets were formed by referring to population data of Leipzig and then (ii) those bins were filled in sequence by subjects who fulfilled the strict exclusion criteria based on the telephone interview. Therefore many subjects, especially in the older age groups were not allowed to participate and it took longer to fill those bins with adequate subjects in order to have true normal data. (iii) Some subjects had to be excluded again based on OCT or other measures when abnormalities or degenerations were found. This procedure (i) to (iii) resulted in a match of the bins with the age and gender distribution of the population of Leipzig at the time stamp of analysis.

### Biometric measurements

#### Height and weight

The mean height of the study population was 173 ± 10 cm (range 151 to 198 cm). The mean weight was 75 ± 17 kg (range 43 to 155 kg). Mean height and weight for women were 166 ± 6 cm and 66 ± 13 kg. Mean height and weight for men were 180 ± 8 cm and 84 ± 16 kg. As this data is self-reported, the data can only serve as an indicator. The body mass index (BMI) was then calculated, resulting in a mean of 25.0 ± 4.6 kg/m^2^ (normal weight) (range 16.8 severe underweight to 46.8 kg/m^2^ adipositas III). Women had a calculated BMI of 24.0 ± 4.2 kg/m^2^ and men had a calculated BMI of 25.9 ± 4.5 kg/m^2^. Taller people had longer eyes (*r* = 0.374, *p* < 0.001), deeper anterior chamber depths (*r* = 0.240, *p* < 0.001) and greater radii of corneal curvature (*r* = 0.325, *p* < 0.001). For regression equations, see Table 8. Body height was associated with several biometric parameters reported; height-adjusted variables are presented in Table 8.

#### Corneal curvature

The mean horizontal radius of curvature of the anterior cornela surface was 7.91 ± 0.26 mm (range 6.92 to 8.74 mm) with its corresponding refracting power being 47.62 ± 1.59 D (range 43.00–54.37 D). The radius was correlated significantly only with AL (*r* = 0.398, *p* < 0.001), but only with modest effect. The mean vertical radius of curvature of the anterior corneal surface was 7.73 ± 0.28 mm (range 6.45–8.59 mm) and the corresponding refracting power was 48.68 ± 1.78 D (range 43.78–58.27 D). The mean radius of curvature of the anterior corneal surface was 7.82 ± 0.26 mm (range 6.81–8.66 mm). The mean horizontal radius of curvature of the posterior corneal surface was 6.65 ± 0.27 mm (range 5.88–8.00 mm) and the corresponding refracting power was −6.03 ± 0.24 D (range −6.80 to −5.00 D). The mean vertical radius of curvature of the posterior corneal surface was 6.29 ± 0.26 mm (range 5.41–6.90 mm) with its corresponding refracting power being −6.37 ± 0.26 D (range −7.40 to −5.80 D). The mean radius of curvature of the posterior corneal surface was 6.47 ± 0.25 mm (range 5.68–7.09 mm). See Table 4 for stratification by gender and age.

Corneal eccentricity (e) as the measure of corneal asphericity was assessed [44]. Mean eccentricity of the anterior corneal surface (e ant) was 0.38 ± 0.19 (range −0.12 to 0.72) and mean eccentricity of the posterior corneal surface (e post) was 0.16 ± 0.36 (range −0.48 to 0.90). Eccentricities for both the anterior and posterior surfaces were mildly associated with age (e ant = 0.56 – 0.004 Age, *r* = 0.340, *p* < 0.001; e post = − 0.41 + 0.013 Age, *r* = 0.439, *p* < 0.001). The eccentricity of anterior and posterior corneal surface was each associated differently with anterior chamber depth. The anterior eccentricity was independent of ACD (*r* = 0.046, *p* = 0.496), i.e., the shape of the surface of the front of the cornea is not associated with ACD. The posterior eccentricity was moderately negatively correlated with ACD (*r* = −0.430, *p* < 0.001): a shallower ACD resulted in eccentricities closer to 1 reflecting an ellipse (e between 0 and 1) or even a parabola(e =1), whereas deeper ACD were associated with a more spherical shape (if e =0 the curve is a circle) [44].

#### Central corneal thickness

Mean central corneal thickness (CCT) was 554 ± 32 μm (range 454–666 μm). CCT was not associated with age (*r* = 0.026, *p* = 0.701), refractive error SE (*r* = 0.039, *p* = 0.568), AL (*r* = 0.116, *p* = 0.087) nor ACD (*r* = −0.018, *p* = 0.793).

#### Axial length

Mean axial length was 23.80 ± 1.05 mm (range 20.89–27.42 mm). Women had shorter ALs (*p* < 0.001) and axial length differed as a function of age (AL = 24.4 – 0.0141 Age), although, the correlation was very weak (*r* = −0.184, *p* < 0.001). Myopic eyes were longer than hyperopic eyes (*r* = −0.674, *p* < 0.001). Longer eyes had a deeper ACD (*r* = 0.623, *p* < 0.001) and a greater ACV (*r* = 0.652, *p* < 0.001). There was an increase in corneal curvature radii with increasing axial length (*r* = 0.398, *p* < 0.001). AL moderately correlated with UDVA (*r* = 0.431, *p* < 0.001). Stratification by age and gender is displayed in Table 4, stratification by refractive category is depicted in Tables 5, 6 and 7.

#### Anterior chamber depth

Mean anterior chamber depth (ACD) was 2.83 ± 0.37 mm (range 1.84–3.74 mm). Men (2.92 mm) had deeper mean ACD than women (2.74 mm) (*p* < 0.001) and in general older people had shallower anterior chamber depths than younger people as a modest negative correlation was found with age (ACD = 3.50 – 0.0155 Age; *r* = −0.555, *p* < 0.001). ACD was moderately associated with refractive error based on SE of subjective refraction (ACD = 2.76 – 0.0938 SE; *r* = −0.519, *p* < 0.001), showing myopic subjects had a greater ACD than hyperopic subjects.

#### Anterior chamber volume

Mean anterior chamber volume (ACV) was 160.1 ± 39.5 mm^3^ (range 71.00–283.00 mm^3^). Men had greater ACVs (*p* < 0.001). Age and ACV were moderately correlated (*r* = −0.519, *p* < 0.001, ACV = 225 – 1.52 Age). The association between ACV and ACD (*r* = 0.900, *p* < 0.001) and the association between ACV and AL (*r* = 0.652, *p* < 0.001) can be described further: subjects with greater ACD (ACV = −108 + 94.7 ACD) and longer eyes (ACV = −420 + 24.3 AL) had greater ACV. ACV was associated with refractive error (*r* = −0.468, *p* < 0.001). Myopic subjects, when stratified by subjected best corrected SE, had the greatest ACV (H < E < M: 125 < 157 < 178 mm^3^). See also stratification by five refractive state in Tables 5, 6 and 7.

#### Retinal thickness

Measurements of retinal thickness were available for 206 subjects. Each OCT scan was manually centred to optimize grid location according to foveal centre, correcting for any possible decentration due to fixation errors. Central retinal thickness, defined here as central foveal subfield thickness (CFST, i.e., mean thickness within the circular central subfield (1 mm diameter, [22])) for the subject group was normally distributed. Minimal central retinal thickness (CRTmin) defined as the thinnest value was not normally distributed. The average across subjects for the CFST was 279 ± 21 μm (range: 227–337 μm). For CRTmin the mean was 232 ± 20 μm (range: 191–317 μm), the median was 230 μm. The mean ± SD of CRTmin and CFST thicknesses in men were 233 ± 20 μm (median 232 μm) and 285 ± 20 μm, respectively. CFST was statistical significantly different for men compared to women (*p* < 0.001). The mean ± SD of CRTmin and CFST thickness in women were 230 ± 20 μm (median 228 μm), and 274 ± 19 μm, respectively. No relationship of CRTmin with gender (Mann-Whitney U test (MW-U); *p* = 0.162,) or age (rank correlation; *r* = 0.13, *p* = 0.06) was found. Although the younger age categories presented with thinner retinas, this comparison was not statistically significant, *p* = 0.403. CRTmin presented with the following median thicknesses separated by gender for the decades investigated, 20–29: male (m):225 μm, female (f):218 μm; 30–39: m: 233 μm, f: 225 μm; 40–49: m: 234 μm, f:232 μm; 50–59: m: 234 μm, f: 228 μm; 60–69: m: 235 μm, f: 231 μm.

When CFST and CRTmin were stratified based on three groups of SE (subjective best corrected), there was no statistically significant difference between CRTmin or CFST between the investigated refractive categories. However the trend presented with slightly smaller CFST and CRTmin values for myopic eyes compared to emmetropes or hyperopic eyes, i.e., longer eyes presented with thinner retinal thickness (CFST = 295–0.65 AL, *r* = −0.033, *p* = 0.634).

### Optics and visual function

#### Refractive error

The mean of the subjective refractive error across the sample was calculated by employing the dioptric distance (DD) to a 0.00 D lens per subject, allowing averaging across the sample. Based on that, the mean refractive error was DD: 1.55 ± 1.63 D (range 0.00–8.38 D); women 1.45 ± 1.59 D; men 1.64 ± 1.67 D.

Mean sphere (based on subjective, right eye) was −0.45 ± 2.05 D (range −8.00 to +6.50 D). Mean cylinder (subjective, right eye) was −0.58 ± 0.61 D (range 0.00 to −4.00 D). Mean SE (objective, right eye) was −0.75 ± 2.06 D (range −9.125 to +6.00 D), see also Fig. 1. The spherical equivalent of the best corrected subjective refraction resulted in −0.69 ± 2.13 D for the right eye (81 emmetropic eyes) and in −0.66 ± 2.09 D for the left eye with a mean best corrected visual acuity of −0.12 ± 0.08 logMAR for either eye respectively.

A weak correlation of sphere (subjective) and age (*r* = 0.356, *p* < 0.001) and spherical equivalent (subjective) and age (*r* = 0.335, *p* < 0.001) suggests a hyperopization for older ages (Table 1). SE was additionally weakly to moderately associated with UDVA (*r* = −0.544, *p* < 0.001), ACD (*r* = −0.519, *p* < 0.001), ACV (*r* = −0.468, *p* < 0.001) and AL (*r* = −0.661, *p* < 0.001). For stratification by gender and age see Table 4, for stratification by refractive state see Tables 5, 6 and 7.

There was a strong correlation between sphere (*r* = 0.978, *p* < 0.001) and spherical equivalent (*r* = 0.979, *p* < 0.001) from respectively subjective and objective refraction. Therefore the values of the subjective refraction were chosen for analysis in the present paper because BCVA was taken as the gold standard.

Inclusion criteria restricted large refractive error, and therefore the reported data are truncated deliberately. 44 % of the study population were myopic (M), 37 % emmetropic (E) and 19 % hyperopic (H). The average uncorrected and corrected visual acuity were +0.25 ± 0.42 logMAR (range −0.26 to +1.50 logMAR) and −0.12 ± 0.08 logMAR (range −0.30 to +0.20 logMAR), respectively.

#### Spherical equivalent and dioptric distance

Additionally to the refractive error summarized above, the difference between the subjective refraction and the existing spectacle lens of a subject was calculated by employing the dioptric distance [23]. For the 218 subjects, the mean dioptric distance found between best visual acuity based refractive correction and habitual correction was 0.50 ± 0.35 D ranging from 0.00 to 1.63 D; women 0.50 ± 0.36 D; men 0.50 ± 0.35 D. There was an increase of need for optimised correction with age, for stratification by age, see Tables 1 and 2.

**Table 2:** “Visual acuity and refractive data stratified by refractive error”

	Myopes	Emmetropes	Hyperopes
UDVA [logMAR]	+0.52 ± 0.43	−0.09 ± 0.09	+0.32 ± 0.32
CDVA [logMAR]	−0.10 ± 0.08	−0.15 ± 0.07	−0.10 ± 0.09
Sphere [D]	−2.01 ± 1.81	0.11 ± 0.34	2.05 ± 1.30
SE [D]	−2.40 ± 1.83	−0.06 ± 0.31	+1.79 ± 1.23
DD to habitual correction [D]	0.52 ± 0.35	0.34 ± 0.26	0.73 ± 0.38
DD to a 0.00D lens [D]	2.47 ± 1.82	0.34 ± 0.19	1.74 ± 1.21
AL [mm]	24.38 ± 1.06	23.54 ± 0.73	22.98 ± 0.79
CRTmin [μm]	231.08 ± 20.07	231.76 ± 20.50	232.51 ± 18.75
CFST [μm]	279.01 ± 20.58	279.49 ± 20.70	279.28 ± 20.67
height adjusted data (for procedure see Table 8)			
AL_adj [mm]	24.32 ± 1.01	23.57 ± 0.65	23.03 ± 0.74
CRTmin_adj [μm]	230.96 ± 19.76	232.03 ± 20.47	233.17 ± 18.89
CFST_adj [μm]	278.10 ± 19.66	279.61 ± 20.18	280.41 ± 19.60

Caption: Data stratified by refractive state based on spherical equivalent (SE) of subjective refraction (mean ± standard deviation) determined by best corrected visual acuity. Data grouped into three refractive states by SE based on the following criteria: hyperopia > +0.50 and myopia < −0.50. Uncorrected distance visual acuity (UDVA) was best for emmetropes, corrected distance visual acuity (CDVA) was relatively equal between groups, mean SE identified that myopia was the highest absolute refractive error of the sample. Dioptric distance (DD) was employed to present the change of the best corrected subjective refraction to the previous corrective state (e.g., spectacle correction if present). Here a value of zero would indicate that former correction and current refraction matched. Based on this, the mean DD between best corrected refraction and current spectacle correction was 0.52D (SD ±0.35) for myopes, 0.34D (±0.26) for emmetropes and 0.73D (±0.38) for hyperopes. To summarise the refractive error present in the sample (218 subjects) by dioptric distance to a 0.00D lens, the group presented with 1.55 ± 1.63 D (range 0.00 to 8.38 D); women 1.45 ± 1.59 D; men 1.64 ± 1.67 D. Stratified by SE, these values for DD were 2.47D (±1.82) for myopic subjects, 0.24D (±0.19) for emmetropes and 1.74D (±1.21) for hyperopes, which is supplied here for comparison with the routinely used measure of SE when stratified into the three groups

In comparison to other studies and with respect to the clinical routine, the SE measure is more commonly used. Using spherical equivalent as a marker, it was investigated how the refractive status was distributed across the study cohort and if improvement of vision by new subjective refraction resulted in changed correction. Interestingly, 118 (54 %) were habitual spectacle wearers, of those 81 (69 %) presented with a change of refraction of over ±0.50 D in one or both eyse established by subjective best corrected refraction in comparison to habitual correction. 100 (46 %) wore no glasses prior to the study and for 46 (46 %) of those a change of refraction of over ±0.75 D in one or both eyes was found. This resulted in 58 % of the study cohort with a change of refraction which might require a need of new or updated spectacle correction (64 % of those already habitual spectacle wearers).

To allow cross-comparison with previous work where the more time-consuming measure of subjective refraction had not been carried out [45, 46], the change of refraction was additionally assessed based on the objective refraction. In order to investigate if a subject required a new refractive correction in comparison to such studies, the change in refractive error is additionally presented here based on the objective refraction measurements carried out. Based on this, the change in objective refraction resulted in 76 (64 %) of 118 spectacle wearers (i.e., 54 % of the study population) with a change of refraction of over ±0.50 D in one or both eyes. 100 (46 %) wore no glasses prior to the study, 16 (16 %) of those were found to be require a change of refraction of over ±0.75 D in one or both eyes; i.e., change to a value of zero, representing no previous habitual correction). Here, 42 % of the study cohort presented a deviation of objective refraction to habitual spectacle correction (83 % of those already previous spectacle wearers). Using dioptric distance as a marker, it was investigated how the refractive status was distributed across the study cohort and if improvement of vision by new subjective refraction resulted in changed correction. Here, 71 (60 %) of 118 (54 %) habitual spectacle presented with a change of refraction of over ±0.35 D in one or both eyes. 101 (46 %) wore no glasses previous to the study, 47 (47 %) of those had a change of refraction of over ±0.50 D in one or both eyes. This resulted in 54 % of the study cohort with a change of refraction which might require a need of new or updated spectacle correction (60 % already habitual spectacle wearers). As can be seen from Table 1, older subjects presented with poorer accuracy of current spectacle lens correction. The deviation of the habitual corrective state to its optimum increased with increasing age. A second group identified with need for improvement of refractive correction was 20–29 years old, where about half a dioptre blur was measured.

#### Accommodation

Mean binocular amplitude of accommodation was 2.65 ± 1.92 D, 2.67 ± 2.07 D in women and 2.63 ± 1.77 D in men. Accommodation was found to differ between age groups and the amplitude of accommodation was progressively less for older ages (Accommodation = 10.2 – 0.158 Age, *r* = −0.826, *p* < 0.001). Detailed binocular amplitudes of accommodation for age groups were 20–29 years 4.32 ± 2.13 D, 30–39 years 3.92 ± 1.86 D, 40–49 years 2.12 ± 0.96 D, 50–59 years 1.07 ± 0.50 D and 60–69 years 1.29 ± 0.67 D. Accommodation had moderate correlations with ACD (*r* = 0.498, *p* < 0.001) and ACV (*r* = 0.484, *p* < 0.001).

#### Contrast sensitivity

**Fig. 2:**
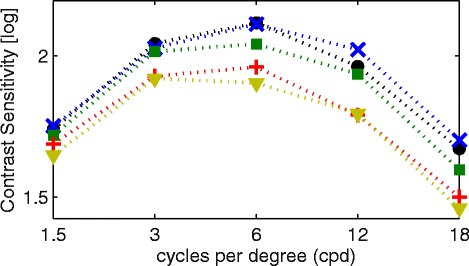
“Contrast sensitivity measured with the Visual Contrast Test System chart and its association with age”. Caption: Contrast sensitivity for all age groups: ● 20–29 years; x 30–39 years; □ 40–49 years; + 50–50 years; ∆ 60–69 years. Gratings examined consisted of spatial frequencies of 1.5, 3, 6, 12 and 18 cycles per degree

**Fig. 3:**
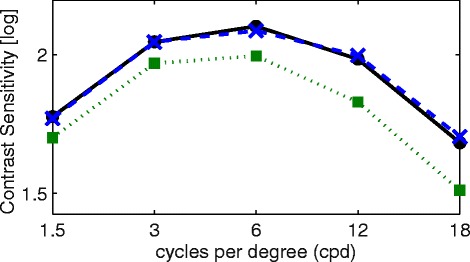
“Contrast sensitivity results for different spatial frequencies stratefied by refractive status”. Caption: Contrast sensitivity for the three refractive states, stratified by spherical equivalent of subjective refraction determined by best corrected visual acuity: ● myopia; x emmetropia; □hyperopia. Gratings examined consisted of spatial frequencies of 1.5, 3, 6, 12 and 18 cycles per degree

Mean contrast sensitivity (log units) in spatial frequencies of 1.5, 3, 6, 12 and 18 cpd was 1.70 ± 0.18, 1.99 ± 0.18, 2.04 ± 0.20, 1.90 ± 0.26 and 1.58 ± 0.27, respectively. For comparison of levels of spatial frequency data, women seemed to have a better contrast sensitivity than men. As can be seen from Fig. 2, contrast sensitivity differed amongst the stratified age groups and was lower at each spatial frequency for older subjects. This association with age was confirmed by the analysis on the basis of the area under the log contrast sensitivity function (AULCSF) curve (AULCSF = 2.29 - 0.005 Age, r = −0.379, p < 0.001). As shown in Fig. 3, hyperopic subjects seemed to have reduced contrast sensitivity. The AULCSF curve was 2.08 ± 0.19 and 31.16 ± 3.19 with linear integration if spatial frequencies were not logarithmic. AULCSF data for women was 2.04 ± 0.18 and for men was 2.09 ± 0.18, this difference was statistically significantly different from zero (*p* = 0.038). Separated by refractive category, myopes presented with an AULCSF of 2.04 ± 0.19, emmetropes with an AULCSF of 2.07 ± 0.17 and hyperopes with an AULCSF of 2.11 ± 0.18, respectively, however there was no significant difference between them. There was no clinical relevant association with AULCSF and best corrected SE (AULCSF = 2.07 + 0.012 SE, *r* = 0.136, *p* = 0.045).

#### NEI − VFQ − 25

The composite score of the NEI − VFQ − 25 can range between 0 and 100, depending on the answers of the subject. Our sample presented with scores ranging from 74 to 100. Most subjects (72 %) had scores over 90. The subscale “General Health” had a mean score of 70. All subscales had a score over 80.

## Discussion

The data presented are part of a multicentre European study on ocular biometric values and visual functions in healthy eyes across the life span. The main purpose of the study is to create a large reference catalogue. This study provides normative data on ocular biometry in a Caucasian adult population of the clinical centre in Leipzig (*n* = 218) aged 21–69 years. In addition to ocular biometric data, we present data on refraction and stature.

### Biometric measurements

#### Height and weight

As expected, taller people had longer eyes, deeper anterior chamber depths and greater corneal curvature radii. This is in agreement with findings of previous studies [4, 7, 45]. Based on previous reports difference in stature was shown to be partially responsible for gender differences found in ocular biometry [7, 47–49]. The current study investigated the effect of body height in association with statistical significant gender differences found for corneal radii, CCT, ACD, ACV, AL and CFST. After adjustment for body height on the basis of a regression model, parameters shown in Table 8 no longer exhibited such gender difference.

#### Corneal findings

Front surface and back surface corneal curvature measured with Pentacam presented four values per subject. The averages are presented in Table 3. Pentacam studies have to take into consideration that corneal power (CP) as given by the built in software employs a refractive index of 1.3375 in order to present a comparable measure to Placido disc systems and allowing for the CP value to be used directly in standard intraocular lens calculations. Instead of the historically used refractive index in keratometry (1.3375), the Pentacam gives more detailed information on the radii and enables CP calculation employing the corneal refractive index of 1.376. Therefore, this study relied on corneal radii when interpreting the curvature data. Front surface corneal radius is an important measure for many clinical investigations, for example fitting of contact lenses. In this study, no age effect on corneal radius was found. A relationship with age was also not found for CP in this study, which is in line with literature. However, former research suggested an association with gender: It has been suggested that women have greater CP (mean difference 0.72 D [50]; mean difference: 0.74 D [40, 51] difference of the means 0.32 D [52]). In terms of radii, the same applied, women were found to have smaller radii than men (mean difference of 0.14 mm [2], 0.11 mm [4] and 0.12 mm (difference of the means, [52]). The results of this study are in line with this, with a mean difference of 0.09 mm and difference of the mean of 0.13 mm. Statistical analysis presented with a gender effect for anterior and posterior corneal radii, see Tables 4 and 8. Height-adjusted mean anterior and posterior corneal radii presented with no further gender effect, see Table 8.

**Table 3:** “Axial length in the literature”

	Year	Method	Axial Length [mm]
Gullstrand		Biometry of enucleated eyes and calculations	24.387
Leipzig	2011	IOLMaster	23.80 ± 1.10
Leipzig_height adjusted data	2011	IOL Master	23.80 ± 0.98
Statistical eye model for normal eyes	2011	IOLMaster	23.67 ± 1.12
The Singapore Malay Eye Study	2010	IOLMaster	23.55 ± 0.05
The Liwan Eye Study	2009	A-mode ultrasound	23.11 ± 0.63
The Meiktila Eye Study	2007	A-mode ultrasound	22.74 ± 0.93
Optical components interactions in emmetropes	2007	A-mode ultrasound	23.34 ± 0.71
The Reykjavik Eye Study	2005	A-mode ultrasound	23.89 ± 1.09
The Los Angeles Latino Eye Study	2005	A-mode ultrasound	23.38 ± 1.01
The Tanjong Pagar Survey	2003	A-mode ultrasound	23.24 ± 0.05

Caption: Axial length values (mean ± standard deviation) for several studies with IOLMaster or ultrasound [2, 4, 7, 47, 50, 51, 55, 56, 89]

**Table 4:** “Corneal biometry, anterior chamber and axial length stratified by gender and age”

	CC R1 ant [mm]	CC R2 ant [mm]	CC R1 post [mm]	CC R2 post [mm]	CCT [μm]	ACD [mm]	ACV [mm^3^]	AL [mm]	CRTmin [μm]	CFST [μm]	CRTmin_adj [μm]	CFST_adj [μm]
									*n* = 206	*n* = 206	*n* = 206	*n* = 206
All (218)	7.91 ± 0.26	7.73 ± 0.28	6.65 ± 0.27	6.29 ± 0.26	554 ± 32	2.81 ± 0.38	160 ± 40	23.8 ± 1.1	231 ± 20	279 ± 21	231 ± 20	279 ± 20
Women (110)	7.85 ± 0.27	7.68 ± 0.27	6.60 ± 0.26	6.26 ± 0.25	549 ± 32	2.74 ± 0.38	149 ± 37	23.4 ± 1.0	230 ± 20	273 ± 19	231 ± 20	278 ± 19
20–29 (24)	7.85 ± 0.30	7.69 ± 0.32	6.62 ± 0.27	6.27 ± 0.28	547 ± 35	3.01 ± 0.34	174 ± 31	23.4 ± 1.0	222 ± 17	271 ± 20	224 ± 16	274 ± 19
30–39 (19)	7.93 ± 0.24	7.73 ± 0.26	6.72 ± 0.24	6.32 ± 0.26	541 ± 41	2.91 ± 0.32	168 ± 33	24.0 ± 0.9	228 ± 22	273 ± 19	229 ± 21	275 ± 17
40–49 (32)	7.78 ± 0.26	7.62 ± 0.23	6.52 ± 0.25	6.17 ± 0.19	554 ± 31	2.76 ± 0.30	144 ± 26	23.5 ± 1.0	233 ± 14	278 ± 17	235 ± 14	282 ± 18
50–59 (19)	7.89 ± 0.31	7.72 ± 0.30	6.59 ± 0.29	6.28 ± 0.29	549 ± 27	2.42 ± 0.22	122 ± 38	23.0 ± 0.8	234 ± 27	274 ± 19	235 ± 26	278 ± 19
60–69 (16)	7.87 ± 0.23	7.72 ± 0.20	6.61 ± 0.24	6.30 ± 0.21	551 ± 21	2.50 ± 0.37	130 ± 32	23.3 ± 0.9	233 ± 20	274 ± 25	236 ± 20	279 ± 24
Men (108)	7.96 ± 0.24	7.78 ± 0.28	6.69 ± 0.28	6.33 ± 0.26	559 ± 32	2.92 ± 0.35	172 ± 39	24.2 ± 1.0	233 ± 20	285 ± 20	232 ± 20	280 ± 20
20–29 (26)	7.94 ± 0.21	7.74 ± 0.22	6.67 ± 0.24	6.27 ± 0.26	559 ± 39	3.18 ± 0.19	196 ± 21	24.4 ± 0.8	231 ± 20	283 ± 21	229 ± 20	278 ± 22
30–39 (20)	7.94 ± 0.30	7.78 ± 0.31	6.65 ± 0.27	6.34 ± 0.34	557 ± 28	3.05 ± 0.19	187 ± 23	24.5 ± 1.1	237 ± 23	289 ± 23	235 ± 23	284 ± 24
40–49 (29)	8.01 ± 0.28	7.83 ± 0.28	6.72 ± 0.28	6.35 ± 0.27	559 ± 31	2.85 ± 0.31	167 ± 41	24.2 ± 1.1	233 ± 19	284 ± 17	232 ± 19	281 ± 17
50–59 (19)	7.95 ± 0.20	7.80 ± 0.21	6.68 ± 0.22	6.35 ± 0.19	560 ± 38	2.81 ± 0.44	160 ± 45	23.9 ± 1.2	233 ± 17	285 ± 20	231 ± 17	280 ± 20
60–69 (14)	7.97 ± 0.19	7.76 ± 0.42	6.78 ± 0.39	6.35 ± 0.19	558 ± 22	2.55 ± 0.30	128 ± 26	23.7 ± 0.7	234 ± 23	280 ± 23	234 ± 23	279 ± 22
Statistical comparison women to men (all)	*p* = 0.001	*p* = 0.008	*p* = 0.015	*p* = 0.028	*p* = 0.025	*p* < 0.001	*p* < 0.001	*p* < 0.001	*p* = 0.162	*p* < 0.001	*p* = 0.903	*p* = 0.324

Caption: Data stratified by gender and age (mean ± standard deviation). Additionally the statistical comparison of each parameter analysed by two sample *t*-test stratified by gender is given in the lower portion of the table

There was a trend in that men had thicker central corneas than women (mean difference 10 μm and difference of the means 10 μm) see Tables 4 and 8, partially this gender effect might be explained by differences in height, see Table 8. There was no effect of age on CCT. This confirms the findings of the Reykjavik Eye Study who found no significant changes in CCT with age, nor any difference between the eyes of men and women. A recent investigation found that men had thicker central corneas than women (difference of the means 5 μm), and suggested that younger subjects had thicker corneas [13].

The eccentricities of the anterior and posterior corneal surface found in this study were in agreement with the findings of an Iran population, where the mean corneal eccentricity was +0.27 ± 0.63 measured with the Pentacam HR [53]. Asgari et al. further discussed a great variety of the normal range of eccentricity between studies, which might be due to different corneal topography devices used. For example, Sicam et al. [54] found mean eccentricities for the anterior and posterior corneal surface of 0.87 ± 0.11 and 0.77 ± 0.17 measured with the Topcon SL-45 Scheimpflug camera.

#### Axial length, anterior chamber depth and anterior chamber volume

Axial length was statistically significantly larger in men compared to women (*p* < 0.001), this had been shown before [47–49]. Height-adjusted axial length presented with no further gender effects, see Tables 8. Stratified into five refractive categories, men presented with statistically significantly larger axial lengths compared to women in all but the low hyperopia category (*p* = 0.160), see also Tables 5, 6 and 7.

**Table 5:** “Corneal biometry, anterior chamber and axial length stratified by refractive error-all subjects”

		CC R1 ant	CC R2 ant	CC R1 post	CC R2 post	CCT	ACD	ACV	AL
Manifest myopia (< −2D)	mean	7.77	7.55	6.57	6.16	552.81	3.11	185.59	25.07
	SD	0.26	0.33	0.36	0.25	34.05	0.24	27.69	1.12
Low myopia (−2D ≤ −0.5D)	mean	7.89	7.73	6.65	6.33	549.02	3.04	179.31	24.20
	SD	0.23	0.25	0.24	0.26	31.35	0.33	35.30	0.90
Emmetropia (−0.5D ≤ ≥ +0.5D)	mean	7.91	7.74	6.64	6.27	554.13	2.83	159.18	23.54
	SD	0.26	0.25	0.25	0.24	34.07	0.32	35.05	0.72
Low hypermetropia (+0.5D ≥ +2D)	mean	8.01	7.84	6.71	6.38	556.86	2.54	135.06	23.41
	SD	0.28	0.28	0.27	0.28	32.38	0.28	35.11	0.79
Manifest hypermetropia (> +2D)	mean	7.90	7.74	6.71	6.32	559.94	2.34	109.76	22.51
	SD	0.30	0.28	0.29	0.27	29.54	0.29	24.78	0.74

Caption: Data stratified by refractive state based on sphere of subjective best corrected vision (mean ± standard deviation). Anterior radii of the cornea (flat: R1 ant, steep: R2 ant) and posterior radii of the cornea (flat: R1 post, steep:R2 post) presented with the following relationship compared for myopic and hyperopic groups: for manifest myopia, the flattest anterior radius (R1) was steeper compared to manifest hypermetropia, the steep counterpart (R2 ant) again was steepest for the myopic group. The corresponding posterior radius presented with a similar change in steepness (again R1 post and R2 post being steepest for myopes). The difference for all four radius parameters was ~0.2 from manifest myopia to manifest hyperopia, reflecting the appearance of a minus lens of the cornea within the optics of the eye. The difference between R1 ant and R2 ant was about 0.2 mm within each refractive category, i.e. for manifest myopia to manifest hyperopia, between R1 post and R2 post this difference for each refractive category was ~ 0.4 mm. Central corneal thickness (CCT) was similar for manifest myopia and manifest hyperopia groups. Anterior chamber depth (ACD) and – volume (ACV) was smaller in hypermetropic subjects. Axial length (AL) was shortest for subjects with manifest hypermetropia. See Tables 6 and 7 for stratification based on gender

**Table 6:** “Corneal biometry, anterior chamber and axial length stratified by refractive error - women”

		CC R1 ant	CC R2 ant	CC R1 post	CC R2 post	CCT	ACD	ACV	AL
Manifest myopia (< −2D)	Mean	7.72	7.50	6.48	6.13	542.81	3.09	180.38	24.64
	SD	0.30	0.28	0.26	0.26	36.93	0.27	29.88	1.07
Low myopia (−2D ≤ −0.5D)	Mean	7.86	7.72	6.65	6.34	551.11	2.87	160.53	23.73
	SD	0.22	0.24	0.22	0.20	33.83	0.38	36.71	0.83
Emmetropia (−0.5D ≤ ≥ +0.5D)	Mean	7.84	7.69	6.59	6.22	551.34	2.76	147.52	23.21
	SD	0.25	0.23	0.25	0.21	33.37	0.32	28.72	0.66
Low hypermetropia (+0.5D ≥ +2D)	Mean	8.01	7.82	6.72	6.38	546.50	2.46	131.70	23.21
	SD	0.25	0.23	0.25	0.21	33.37	0.32	28.72	0.66
Manifest hypermetropia (> +2D)	Mean	7.67	7.53	6.50	6.18	557.13	2.20	96.13	21.98
	SD	0.19	0.15	0.20	0.23	33.88	0.15	9.08	0.59

Caption Data stratified by gender (female) and refractive state based on sphere of subjective best corrected vision (mean ± standard deviation). Women presented with steeper anterior radii of the cornea (flat: R1 ant, steep:R2 ant) and steeper posterior radii of the cornea (flat: R1 post, steep:R2 post) compared to men (see Table 7)

**Table 7:** “Corneal biometry, anterior chamber and axial length stratified by refractive error - men”

		CC R1 ant	CC R2 ant	CC R1 post	CC R2 post	CCT	ACD	ACV	AL
manifest myopia (< −2D)	Mean	7.82	7.60	6.67	6.20	562.81	3.12	190.81	25.51
	SD	0.20	0.37	0.42	0.22	28.62	0.21	25.17	1.01
low myopia (−2D ≤ −0.5D)	Mean	7.92	7.74	6.65	6.33	547.50	3.16	193.04	24.55
	SD	0.23	0.26	0.25	0.31	30.00	0.22	27.54	0.79
emmetropia (−0.5D ≤ ≥ +0.5D)	Mean	7.99	7.81	6.69	6.33	557.30	2.90	172.43	23.90
	SD	0.24	0.25	0.25	0.26	34.96	0.30	37.13	0.62
low hypermetropia (+0.5D ≥ +2D)	Mean	8.02	7.87	6.69	6.38	569.81	2.63	139.25	23.66
	SD	0.27	0.28	0.25	0.23	37.76	0.29	31.91	0.85
manifest hypermetropia (> +2D)	Mean	8.11	7.94	6.89	6.44	562.44	2.45	121.89	22.99
	SD	0.23	0.23	0.23	0.26	26.94	0.33	28.36	0.49

Caption: Data stratified by gender (male) and refractive state based on sphere of subjective best corrected vision (mean ± standard deviation). Men presented with flatter anterior radii of the cornea (flat: R1 ant, steep:R2 ant) and flatter posterior radii of the cornea (flat: R1 post, steep:R2 post) compared to women (see Table 6)

In the present study, axial length was slightly larger (men: 24.18 ± 1.01 mm; women: 23.41 ± 0.98 mm) than reported in previous work, e.g., the Reykjavik study [47, 52] (men: 23.74 ± 1.0 mm; women: 23.20 ± 0.98 mm), the Zagreb study (men: 23.49 ± 0.75 mm; women: 23.18 ± 0.67 mm) [55] and the EPIC-Norfolk Eye Study (men: 23.80 ± 1.16 mm; women: 23.39 ± 1.15 mm) [15]. However, the Zagreb study measured only emmetropic subjects and Foster et al. included 52 % hyperopic subjects. A Belgian dataset measured 23.67 ± 1.12 mm [56]. Eysteinsson et al. [47] although men in our study were taller, the main difference in AL observed in Table 3 may be related to the fact that our subjects were measured with the IOL Master. See Table 3 for comparison of different studies, where IOL Master values for AL were higher on average than ultrasound measurements.

Studies by Lam et al., Santodomingo − Rubido et al. and Sheng et al. have shown the IOL Master to be more repeatable than ultrasound for axial length measurement [57–59]. Sheng et al. showed that the repeatability of the IOL Master was excellent regardless of the experience of the observer. Therefore, we chose to measure the axial length with the IOL Master. Table 2 shows that the axial length measured with the IOL Master is slightly longer than the A − scan ultrasound axial length. (This was also shown by Sheng et al.) The differences in axial length between the listed studies may be due to differences in races in population based samples of biometrical data.

Considering different races, AL of Caucasian eyes in this study were longer than East Asian eyes (AL 23.55 mm) measured with IOL Master [4]. In general, AL was associated with ACD and ACV, with an increase in ACD resulting in an increase in ACV. AL decreased with increasing age and women had smaller values, which was in agreement with other findings [2, 4, 47, 50]. However, the current findings established only a low association of age or gender with AL. The decrease of ACD and ACV with age resulted mainly from an increase in lens thickness [60]. In this sample, some association of SE was established with ACD (*r* = −0.519), ACV (*r* = −0.468) and AL (*r* = −0.661). This is contradictory to previous findings where no correlation was found between SE and ACD (*r* = −0.13, *p* = 0.42) [61]. The previous results were based on myopic subjects (0.00 to −14.88 D), whereas the current investigation included a range of hyperopic to myopic subjects (+6.00 to −9.13 D). Gender differences in the current study observed for ACD and ACV were accounted for by body height adjustment, see Tables 4 and 8.

**Table 8:** “Selected gender effects adjusted for by body height”

Variable under investigation	Statistical significant difference male (m) versus female (f)	Regression analysis	Variable after adjustment for body height based on regression model
men: *n* = 108			
women: *n* = 110			
Mean anterior corneal radius (CCRant)	m: 7.87 (SD 0.25); f: 7.77 (SD 0.26); *p* = 0.003	CCRant = 6.37 + 0,00837 Height	m: 7.82 (SD 0.24); f: 7.83 (SD 0.26); *p* = 0.701
Mean posterior corneal radius (CCRpost)	m: 6.51 (SD 0.24); f: 6.43 (SD 0.25); *p* = 0.014	CCRpost = 5.26 + 0,00699 Height	m: 6.46 (SD 0.23); f: 6.48 (SD 0.24); *p* = 0.591
Central corneal thickness (CCT)	m: 558.7 (SD 32.3); f: 548.7 (SD 32.0); *p* = 0.023	CCT = 532 + 0.128 Height	m: 557.3 (SD 32.3); f: 549.2 (SD 32.2); *p* = 0.064
Anterior chamber depth (ACD)	m: 2.92 (SD 0.35); f: 2.74 (SD 0.38); *p* < 0.001	ACD = 1.25 + 0,00912 Height	m: 2.86 (SD 0.35); f: 2.81 (SD 0.38); *p* = 0.314
Anterior chamber volume (ACV)	m: 171.6 (SD 39.2); f: 148.9 (SD 36.7); *p* < 0.001	ACV = − 43.0 + 1,17 Height	m: 163.9 (SD 38.6); f: 157.8 (SD 36.8); 0.235
Axial length (AL)	m: 24.16 (SD 1.01); f: 23.44 (SD 0.97); *p* < 0.001	Axial length = 17.0 + 0.0393 Height	m: 23.88 (SD 0.97); f: 23.72 (SD 0.98); *p* = 0.219
Central foveal subfield thickness (CFST)	m: 284.6 (SD 20.3); f: 273.9 (SD 19.4); *p* < 0.001	CFST = 182 + 0.562 Height	m: 280.4 (SD 20.3); f: 277.7 (SD 19.2); *p* = 0.324
Men: *n* = 103			
Women: *n* = 103			
Minimal retinal thickness (CRTmin)	m: 233.4 (SD 20.1) median 232.0; f: 229.8 (SD 19.7) median 228.0; *p*(MW-U) = 0.162	CRTmin = 194 + 0,216 Height	m: 232.1 (SD 20.1) median: 230.6; f: 231.4 (SD 19.5) median 229.4; *p* (MW-U) = 0.903
Men: *n* = 103			
Women: *n* = 103			

Caption: Mean data stratified by gender for men (*n* = 108) and women (*n* = 110) for corneal radii, CCT, ACD, ACV, AL and retinal thickness measured as CFST and CRTmin. All but CRTmin presented with statistically significant gender effects

Association of respective variables with body height was investigated and adjusted based on a regression model where variable_new = variable_old –regression function + mean (variable_old). After adjustment for body height, all investigated variables presented with no gender effects, therefore differences in stature between men and women may explain some of the differences in the biometric data reported

#### Retinal thickness

Retinal thickness was investigated using CFST and CRTmin. Measured within the central 1 mm diameter area of the retina, statistically significant smaller CFST was found for women compared to men, see Table 8. This confirmed previous findings for CFST on the same OCT device [62, 63] by Wagner-Schuman et al. who established 265 ± 23 μm for men and 254 ± 19 μm for women; *p =* 0.0086 [64]. In cross-reference, Wagner-Schuman and colleagues reviewed several other studies on various OCT devices which also found sex-related differences in retinal thickness with smaller thicknesses for women [64]. In the past, healthy subjects have been investigated on the OCT device of the current study [54, 62]. Wolf-Schnurrbusch et al. [65] in a comparative study with Spectralis SD-OCT Grover et al. found a mean CFST of 271 ± 20 μm [63]. In an earlier study mean CFST was 270 ± 23 μm (men 274 ± 23 μm; women 266 ± 22 μm) based on the small sample size the gender effect had not been found significant (*p* = 0.1) [62]. The CFST data for Caucasians (*n* = 28) in the Grover sample of 50 healthy subjects (273 ± 21 μm) was slightly smaller than the findings of the current investigation [62]. Wolf-Schnurrbusch on the other hand established a CFST of 289 ± 16 μm (right eye) across their sample of 20 subjects [65]. The current study presents normative retinal thickness data on 206 Caucasian subjects and it can therefore be assumed that 279 ± 21 μm is more representative of the population.

One could expect that myopic eyes, which are longer, to have thinner retinal thickness, but this trend apparent in the data was not confirmed statistically. Whereas other studies based on larger subject numbers found in fact that central macular thickness is greater in myopic eyes. The work of Choovuthayakorn et al. demonstrated an increasing CFST and decreasing inner and outer subfield thicknesses with greater axial length [66]. The UK Biobank Study found a mean CFST of 265 ± 23 μm and showed that CFST was positively correlated with greater myopia (*p* < 0.001) [67].

### Optics and visual function

#### Refractive error

In the present study, subjects tended to be more hyperopic in older age groups (SE (subjective) in right eyes of 40–49 year olds: −0.86 ± 2.06 D; 50–59: +0.31 ± 1.92 D; 60–69: +0.45 ± 1.97 D), see also Fig. 1 and caption of Table 1. Nevertheless, the differences in refractive error amongst the sample age groups do not necessarily suggest a change as a function of age. The hyperopic shift resulted in more emmetropic eyes in the 40–49 years decade, followed by a higher percentage of hyperopic eyes from 50 years onwards. This is in line with previous findings. In the Gutenberg Health Study (GHS) myopia was present in 35 % of the study sample and hyperopia in 32 %, with refractive errors ranging from −21.50 to +13.88 D [46]. Wolfram et al. found a higher prevalence of myopia in younger age groups, with a hyperopic shift up to the age of 69 years for their population of 35 to 74 years of age. In a Spanish study population aged 40 to 79 years the prevalences of myopia and hyperopia were 25 % and 44 %, respectively. Myopia did not change significantly with age but hyperopia increased with age [68]. This trend towards hyperopia by increasing age was confirmed by a Norwegian study, whose study population was between 38 and 87 years old [12]. British adults aged 48 to 88 years presented with refractive errors of 27 % myopia and 52 % hyperopia [15]. In a recent meta-analysis of European refractive error studies, the prevalence of myopia and hyperopia was 30.6 % and 25.2 %, respectively [69]. They also reported a hyperopic shift in the older age groups. Several studies with East Asian subjects reported more myopic cohorts compared to the present study, but the general trend also was a hyperopic shift towards old age [2]. High myopia in young Chinese reported recently [70, 71] is not yet part of those subjects; this may influence this observation in future. The present data included age groups from 20–69 years and may have examined more subjects with a higher level of education, which has been associated with a more myopic refraction [72], and thus the prevalence of myopia may be higher in the current investigation ((M) 44 %, (E) 37 %, (H) 19 %). However, the association of the level of education and myopic refraction could not be confirmed in our study sample. Furthermore, Williams et al. used refractive error categories, where emmetropia ranges from −0.74 to 0.99 D, which differed from ours as defined in the methods section. This resulted in a higher prevalence of emmetropia of 43.5 % in the meta-analysis and the results are not useful for direct comparison.

Grouped by gender and age, the spherical equivalent for the cohort in this study is presented in Table 1. A higher prevalence of hyperopia was seen in older compared to younger persons also when separated by gender. Similarly to the presented findings, older women were more hyperopic than men of the same age in American Latino [50], Northern European [13, 47] and Singaporian Chinese subjects [2].

Table 4 shows subjects separated by gender and age decade. This allowed interpretation of ocular biometric values (ACD, ACV, AL, CC and CCT) in separate categories and therefore enabled direct comparison. Tables 5, 6, and 7 presents the same data stratified by refractive state (based on sphere of best corrected subjective refraction), here it is clearly visible that parameters are dependent on refractive status. Myopic subjects compared to manifest hypermetropic subjects presented with steeper anterior and posterior corneal radii, had slightly thinner corneas (CCT) on average and presented with much longer mean anterior chamber depths (ACD), as well as bigger anterior chamber volume (ACV), and their axial length (AL) was longer, see Tables 5, 6 and 7 for additional stratification based on gender.

#### Spherical equivalent and dioptric distance

This study was able to perform an accurate comparison between best − corrected subjective refraction and habitual corrective status, e.g., old glasses. Commonly, the spherical equivalent (SE, sphere plus half of cylinder) is used to compare measures of refraction. However, this is unsuitable when comparing the change in power of two spherocylindrical lenses accurately, as SE does not take the axis into account. Therefore, to display the difference between new refraction to previous correction of a subject, the dioptric distance between both lenses was calculated [23]. The dioptric distance is a helpful tool, especially as it allows accurate grouping of such changes across the population. Future observations may therefore benefit from accurate calculations using dioptric distance.

There was a difference between the state of undercorrection depending on reporting objective (42 %, 83 % of which were previous spectacle wearers) or subjective best corrected data (58 %, 64 % of which were previous spectacle wearers), specifically for the lower refractive errors of non-spectacle wearers (16 % objective versus 46 % subjective). This might be due to the repeatability and validity of the autorefractor used (Humphrey Automated refractor/Keratometer (HARK) 599), this has to be investigated separately. The objective data (S: −0.51D ± 2.02; SE: −0.75D ± 2.1) and subjective data (S: −0.44D ± 2.04; SE = −0.73D ± 2.1) across the whole subject group presented with similar refractive error and SD over all. Previously it was shown that the between-visits-repeatability for all refractive error measurements were <0.75 D and the mean difference between the subjective refraction and the HARK autorefraction for spherical equivalent was statistically significant under non-cycloplegic conditions (−0.90 D, *P* < .0001) and cycloplegic conditions (−2.05 D, *P* < .0001) [73]. Thus, this may have affected specifically the reporting of low refractive errors of previous non-spectacle wearers. Therefore it can be concluded that the subjective best corrected results of the current study represent the true state of the sample investigated. The Gutenberg Health Study presented autorefraction data (HARK) and compared this to self-reported information on the current spectacle or contact lens correction worn and found that 3.5 % of subjects who were in need of refractive correction did not previously possess one based on the criteria of binocular myopia or hyperopia [46]. This is much lower than the state of undercorrection in non-spectacle wearers (16 % based on autorefraction; 46 % based on subjective refraction) in the current investigation even though the present study included a higher percentage of emmetropic subjects (37 vs. 33 % GHS). Additionally, the current study is able to report on the clinical significant change in refraction of spectacle wearers (based on autorefraction: 64 %, based on subjective refraction: 69 %). The Blue Mountains Eye Study stated that undercorrected refractive error was present in 10.2 % of the study population [45]. But they investigated initial visual acuity in comparison with the subject’s habitual correction and defined undercorrection as an improvement of ≥ 10 letters (two lines on the logMAR chart), after refraction in subjects with a presenting visual acuity <45 letters. The high percentages for improvement of correction of refractive error found in this healthy cross-section might have implications on visual acuity for driving, quality of visual performance for the work force and quality of life parameters based on vision. This suggests that the population might benefit from regular eye examinations and vision testing.

#### Visual acuity

Uncorrected and corrected visual acuity were +0.25 ± 0.42 logMAR (−0.26 to +1.50 logMAR) and −0.12 ± 0.08 logMAR (−0.30 to 0.20 logMAR), respectively. Divided into age and gender no differences were found. Visual acuity is therefore only weakly correlated with age (*r* = 0.202). A weak association with age was found in an older Chinese population (40 to over 75 years) (*r* = 0.390) [74], which might be due to older age and larger percentage of vision problems which specifically had been excluded from the present study. No association was found between visual acuity and education in line with findings by Xu et al. [74].

#### Accommodation

Cause for the reduced ability to accommodate with increasing age is a progressive age − related loss of elasticity of the lens capsule, nuclear sclerosis or a lens thickening by lifelong growth of the lens [75], [1]. For this reason accommodation presented with moderate correlations with ACD (*r* = 0.498, *p* < 0.001) and ACV (*r* = 0.484, *p* < 0.001) as a thicker lens influences ACD and ACV, i.e., they are reduced, and in turn this is associated with a decreased ability to accommodate. Yuan et al. [76] reported that ACD decreased significantly with accommodation compared to the non-accommodative condition.

#### Contrast sensitivity

Mean contrast sensitivity (log units) for different spatial frequencies was in line with other studies.

With an increase in spatial frequencies employed (1.5 to 18 cpd, see above) contrast sensitivity increased to its peak at 6 cpd followed by a decrease to its lowest value at 18 cpd [77], [78]. This was expected as contrast sensitivity in the normal human eye peaks at a level of 6 cpd.

The present study was able to establish that contrast sensitivity decreased with age at each spatial frequency based on a larger number of subjects across the life span (20–69 years of age), see Fig. 2. Additionally, pupil diameter in the given population decreased with age (*r* = −0.388, *p* < 0.001), which may in turn have an influence on contrast sensitivity [79]. In comparison with data obtained using the Optec 6500 Functional Acuity Contrast Test (Stereo Optical Company, Chicago, IL), the present study found lower contrast sensitivity measures for lower spatial frequencies (1.5, 3, 6 cpd) whereas for higher spatial frequencies higher contrast values were found [77]. In a different study, the contrast sensitivity function of an older population (40–64 years) was generally lower [80]. However, Hashemi et al. employed the CSV − 1000 (VectorVision, Greenville, OH) and it can generally be noted that the VCTS − 6500 (Vistech, Dayton, OH) produces higher scores than the former apparatus [81]. Note that the younger population tested there also presented lower scores in comparison to the present findings. Contrast sensitivity has been shown to be influenced by refractive error, with myopes showing lower contrast sensitivity values than hyperopes [80]. The present investigation, however, found hyperopic eyes with lower contrast sensitivity than myopic eyes, but this is due to increasing hyperopia with age in the population and therefore age is the predominant factor and not hyperopia, see Figs. 2 and 3 for cross-reference.

The concept of computing the area under the log contrast sensitivity function (AULCSF) provided the advantage of giving one number per subject containing information from all spatial frequencies [82, 83]. This enabled comparison with other measures, additionally serving as a baseline for future studies. Another German study using the functional acuity and contrast chart (FACT) as part of the Contrast Sensitivity Tester 1800 (CST 1800; Vision Science Research Corp.; San Ramon, CA), which is comparable to the VCTS chart, found a decrease in contrast sensitivity (AULCSF) in the older age group (50–69 years) compared to the younger age group (21–47 years) [84]. With the OPTEC 6500 device the relationship of the AULCSF with age has been shown to be *r* = 0.57 (photopic) and *r* = 0.54 (mesopic) where a reduction of contrast sensitivity is associated with increasing age [85].

#### NEI VFQ − 25

Most subjects (72 %) had composite scores over 90. It can be concluded that subjects interviewed in our study are content with their vision and therefore think of their vision as “good”. The subscale “General Health” presented with a mean score of only 70, although the subjects had no chronic disease or any health problems. The questionnaire can be considered a subjective tool and therefore subjects may underestimate their health. All subscales have a score over 80. In comparison, overall lower scores were found for both the English and Spanish speaking groups in a healthy and visually normal subsample of the Latino Eye study [87]. Data on visually impaired Chinese subjects showed a clear association of lower scores with severity of impairment [86]. Another German study examined 511 subjects with good eye health and found a similar composite score of 91.6 ± 7.1 and a higher score for general health (79.9 ± 17.4), and based on their subscales they were able to establish that the subjects had good eye health. They concluded that as a screening tool the VFQ − 25 was not specific or sensitive enough to detect subjects with eye conditions from a random population [88].

Possible shortcomings of our study were the relatively small sample size, limited by the various measurements carried out, the non-randomized study population and - although intended - the missing ethnic diversity, as the study presented results for an exclusive German cohort without other Caucasian subgroups.

The study recruited subjects of all levels of education. In the analysis there was no correlation of the level of education with any result of the examinations found. This may have been due to small numbers per education level, as previous research showed a link between education and refractive status, i.e., myopia was linked to higher education levels [72].

## Conclusion

The data obtained present an overview of the average ocular biometry within the population of Leipzig, Germany. The large subset of parameters established for each subject allows comparison between datasets providing the background for creation of a database. This enables cross-referencing to determine associations between parameters for healthy eyes. The reference values established therefore permit multiple comparisons with patient data for further investigations, especially in view of the correlations established as part of this study.

In this paper, biometrical data based on in − vivo measurements of healthy German eyes are presented and compared stratified by age, gender and refractive status. This resulted in the following conclusions: a decrease with age was found for anterior corneal curvature, anterior chamber depth, and anterior chamber volume. The spherical equivalent was more hyperopic with age. A decrease with age was found for accommodation and contrast sensitivity. Greater anterior corneal curvature, greater anterior chamber depth and greater anterior chamber volume were established for hyperopic subjects than for myopic subjects.

We verified relationships or differences between those parameters based on our data and in reference to the current literature. Our dataset is useful for future work on either a smaller number of subjects or a selected patient group, as data can be directly compared with each aspect given in our study, allowing other research to extract answers for their data without having to establish an age correlated normal sample themselves. In future, with aid of this data existing statistical eye models can be updated [56]. This data of strictly controlled eyes on a multitude of biometric reference parameters in the same eye based on gold-standard techniques, serves as starting points for disease prevention as well as a reference for health policy and practice. Together with detailed information on current habitual correction versus new corrective lenses based on best corrected visual acuity, it provides background to the goal of extending good functional vision into old age.

## Abbreviations

ACD, anterior chamber depth; ACV, anterior chamber volume; AL, axial length; AULCSF, area under the logarithmic contrast sensitivity function; BCVA, best corrected visual acuity; BMI, body mass index; C, cylinder; CC, corneal curvature; CC R ant, mean anterior corneal radius; CC R post, mean posterior corneal radius; CC R1 ant, flat anterior corneal radius; CC R1 post, flat posterior corneal radius; CC R2 ant, steep anterior corneal radius; CC R2 post, steep posterior corneal radius; CCT, central corneal thickness; CDVA, corrected distance visual acuity; CFST, central foveal subfield thickness; CP, corneal power; cpd, cycles per degree; CRTmin, minimal central retinal thickness; DD, Dioptric distance; E, Emmetropia; e, Corneal eccentricity; e ant, eccentricity of anterior corneal surface; e post, eccentricity of posterior corneal surface; f, female; H, hyperopia; logMAR, logarithm of the minimum angle of resolution; M, myopia; M, male; NEI-VFQ-25, National Eye Institute visual functioning questionnaire 25; OCT, optical coherence tomography; S, sphere; SD, standard deviation; SE, spherical equivalent; UDVA, uncorrected distance visual acuity; VCTS, visual contrast test system
